# A cognitive inquiry into similarities and differences between translation and paraphrase: Evidence from eye movement data

**DOI:** 10.1371/journal.pone.0272531

**Published:** 2022-08-05

**Authors:** Xingcheng Ma, Tianyi Han, Dechao Li

**Affiliations:** 1 School of Foreign Languages, Southeast University, Nanjing, China; 2 Department of Chinese and Bilingual Studies, The Hong Kong Polytechnic University, Hong Kong, China; The Chinese University of Hong Kong, UNITED KINGDOM

## Abstract

Intralingual translation has long been peripheral to empirical studies of translation. Considering its many similarities with interlingual translation, also described as translation proper, we adopted eye-tracking technology to investigate the cognitive process during translation and paraphrase, an exemplification of intralingual translation. Twenty-four postgraduate students were required to perform four types of tasks (Chinese paraphrase, English-Chinese translation, English paraphrase, Chinese-English translation) for source texts (ST) of different genres. Their eye movements were recorded for analysis of the cognitive effort and attention distribution pattern. The result demonstrated that: (1) Translation elicited significantly greater cognitive efforts than paraphrase; (2) Differences between translation and paraphrase on cognitive effort were modulated by text genre and target language; (3) Translation and paraphrase did not differ strikingly in terms of attention distribution. This process-oriented study confirmed higher cognitive efforts in inter-lingual translation, which was likely due to the additional complexity of bilingual transfer. Moreover, it revealed significant modulating effects of text genre and target language.

## Introduction

In his famous classification of translation, Jakobsen [1] defines three types of translation, namely, interlingual translation, intralingual translation and intersemiotic translation, which has been quoted ubiquitously as one of the fundamental statements on central features of translation [2]. Intralingual translation, according to Jakobsen’s definition, can be understood as a rewording or paraphrasing, that is, expressing approximately the same meaning in “different forms of the same language [3]”. Scholars have listed a range of situations in which intralingual translation facilitates communication, for example, reformulating a legal document in plain language for a lay readership, new translating of classics to improve its comprehensibility, and easy-readers for children [3, 4]. However, despite its wide practice and its family resemblance to interlingual translation, the bulk of translation studies are devoted to inter-lingual translation and intra-lingual translation has remained an under-explored area of research, in particular, intra-lingual translation has seldom been incorporated into empirical-experimental paradigms.

This study aims to investigate the cognitive processing of interlingual translation and paraphrase (a representative variant of intra-lingual translation) via examining the student translators’ cognitive effort and attention distribution. In the present study, paraphrase is used in its narrower sense, referring to rewording or reformulation in the same language. Thus, it can be differentiated from paraphrase adopted in translation and interpreting as semantic [5] or reformulation strategy [6], which consist of explaining the meaning of a source language item when there is no suitable correspondent in target language.

Based on eye movement data, we focus on student translators’ cognitive processing during translation and paraphrase in both their first (L1) and second language (L2). Such a comparative approach allows us to gain insight into the effect of translation mode on translators’ cognitive mechanism. In addition to the potential mode-induced difference in cognitive processing, we explored whether and how this mode effect is affected by text genre and target language. These explorations are expected to help identify the similarities and differences between inter-lingual and intra-lingual translation from a cognitive perspective.

## Research background

### Paraphrase as a variant of intra-lingual translation

Despite its widespread use in linguistic mediation, intralingual translation does not have a unified definition and seems to be “a broad cluster concept [3]”. Intralingual translation is an interpretative act which expressed the same meaning in different forms of the same language [7], thus can be understood as “rewording” or “paraphrasing”. Although intralingual transfer was implicitly referred to as a less prototypical form of translation and often considered as a peripheral area in translation studies [1, 4], this practice has a long history and includes a variety of individual subtypes such as respeaking, localization, rewriting and adaptation [3], with a set of synonymous names (paraphrasing, rewording, reformulation). According to Pillière [8], the somewhat loose definition of intralingual translation by Jakobsen enables researchers to interpret it as covering a wide spectrum of possibilities. However, this broad range of concepts may also entail problems of definition. For instance, paraphrase, if understood in a broader sense, refers to linguistic operations in both intra- and inter-lingual condition. It has often been taught and practiced in translation and interpreting as a coping or emergency strategy for source items which do not have target correspondents [5]. Thus, to narrow the research scope for concept operationalization in the current study, we focus on paraphrase in intra-lingual setting which is defined as “reformulating the same meaning by using different wording, phrasing and structures in the same language”. To achieve this, the paraphraser must first capture the gist of what was originally stated [9] and refrain from copying too much from the ST or quoting verbatim.

The motivation for choosing paraphrase as an exemplification of intra-lingual transfer is grounded in translator and interpreter training. In pedagogical context, paraphrase exercise in the same language has been applied to training students’ abilities to express the original text’s semantic completeness in different lexical and syntactic structures [10]. Additionally, by practicing paraphrase, students can learn to overcome linguistic barriers and free themselves from surface structures in target delivery [4, 11], which is quite helpful when there is no direct equivalence between source language and target language. For example, translators may use paraphrase when dealing with idiomatic expressions [12, 13].

### Similarities and differences between translation and paraphrase

Previous studies introduce some similarities and differences between paraphrase and translation, which forms the basis for a comparative analysis. According to Malakoff and Hakuta [14], paraphrasing is “particularly relevant to translation because of potential parallels with translation.” First, in terms of the skill components, paraphrase and translation both require the ability to extract meaning from an utterance and to rephrase the equivalent meaning in another utterance. Comprehension and reformulation are necessary in both tasks during which the original messages are encoded, extracted and expressed into different words [15]. Second, in terms of processing stages, the cognitive process of translation and paraphrasing can be conceptualized as a sequence of at least three phases: orientation, drafting and revision [16]. Both tasks are recursive in nature, as characterized by overlapping steps planning, writing and editing [17]. Lastly, as summarized by Whyatt [3], the strategies used in paraphrase largely overlap with those applied in translation. For instance, Bhagat and Hovy [18] listed a range of strategies for paraphrasing a sentence by lexical change which are comparable to those used in translation.

Despite the above similarities, debates continue on whether the cognitive effort required for paraphrase is less than that for translation. There are two conflicting schools of thought on this issue. One school argue that translation and paraphrase, which are distinguished merely by the language mode, involve the same process [19]. In psycholinguistic terms, translation is often regarded as an extreme case of bilingual activity featured by continuous language selection and inhibition process [20], whereas in paraphrase there is no additional complexity of code-switching. Thus, without the burden of language transfer, paraphrase is supposed to be cognitively less demanding than translation. In contrast, the other school believes that paraphrase is not necessarily easier than translation. It is a complex meta-linguistic task which requires keeping semantic completeness of the original text by using different lexical and syntactic resources [10, 11]. Despite the absence of cross-language interference, this process of searching for different lexical and structural forms to express the same meaning is quite similar to that in interlingual translation [4]. And the lack of synonyms or equivalent expressions may increase the difficulty of paraphrase [14, 21]. As is often the case when conflicting views are held, there may be a kernel of truth in both of them. It would be premature to draw a conclusion unless more empirical data are obtained.

### Cognitive explorations of translation and paraphrase

De Groot [22] proposed three approaches to the cognitive study of translation, namely: 1) studying the translation of words and sentences, 2) studying text translation by manipulating input characteristics and 3) comparing translation with similar tasks. Here, similar task refers to activity that contains many of the same components (sub-processes) of the translation task. If the translation task and the comparison task share a range of overlapping features, but differ in one particular component or sub-process, differences between the tasks may be attributed to the component/sub-process that is present in one task but absent in other. Thus, this approach, which is reminiscent of the subtraction method in cognitive psychology [23], is believed to be helpful for identifying the specific demands of certain task modes and the relations between the two tasks.

Based on this comparative approach, several empirical-experimental studies have been carried out to reveal how far paraphrase overlaps with translation in terms of process and products. Kajzer-Wietrzny et al [24] combined corpus linguistics, key-logging and eye-tracking to investigate whether stylistic simplification, a frequently hypothesized indicator of translation universal, was equally present in the end products of paraphrase and translation and how this tendency was related to the level of translation expertise. Whyatt et al [25] reported a comparative study of decision-making process in paraphrase and translation by collecting user activity data (UAD) via key-logging, eye-tracking and screen recording. Their study is part of a ParaTrans project which aims to reveal to what extent paraphrase and translation are cognitively similar or different by focusing on several aspects, for example, the task time in the two modes, relations between real-time decision-making and translation expertise and the manifestation of simplification or explicitation.

To the best of our knowledge, empirical-experimental research on cognitive processing during English/Chinese intra-lingual translation are still few and far between. Little knowledge has been obtained on the cognitive process of paraphrase into Chinese or English as compared to English-Chinese and Chinese-English translation. In addition to the issue of language combination, several aspects regarding the translator or paraphraser’s cognitive processing have been under addressed. First, eye movement measures need be enriched in reflect multiple dimensions of processing. For instance, Kajzer-Wietrzny et al [24] merely used dwell time to indicate the cognitive effort allocated to different textual regions. It is essential to further exploit the advantage of eye-tracking in recording and describing cognitive behaviors by adopting eye movement measures reflective of not only overall cognitive effort but also attention dividing mechanism. Second, statistical analysis with greater explanatory power is required to compensate for the generally small sample size and high individual variations in empirical translation studies. Results primarily derived from descriptive statistics (e.g., mean sentence length, mean processing duration) in Kajzer-Wietrzny et al [24] and Whyatt et al [25] may fail to accommodate for differences within participants or STs. Lastly, participants’ behavioral data in terms of eye movements or key-logging might be co-determined by multiple factors, thus factors that potentially modulate the cognitive processing of paraphrasing and translation, such as text genres, translation directionality (L1 translation vs. L2 translation), and language mode (paraphrasing in L1 vs. paraphrase in L2) need be considered in experimental design. These, unfortunately, have not been examined as variables of interest in previous studies [24, 25]. Translating in non-native language, also known as inverse translation, has become a widely practiced reality around the world [26], which calls for more systematic research on the effect of directionality during intra- and interlingual translation. As for genres, it is believed that texts of different genres are characterized by different styles, structures and contents, which may influence the identification and solution of translation problems [27].

With a focus on real-time viewing behavior, this study adopts a comparative approach to the cognitive processing in paraphrase and translation. Further, it attempts to testify the modulating effect of text genre and target language on paraphrase and translation processes. Guided by a data triangulation method, we primarily use eye-tracking to capture the participants’ viewing behavior in combination with their retrospective protocols and quality of target output. Eye-tracking has become increasingly popular as a quantitative research method in translation process research. Measurements of the duration and location of the fixations allow for observation and analysis of how information was retrieved, comprehended and integrated, which enables researchers to make inference on people’s cognitive processing underlying their eye movement behaviors [28].

Based on the above discussions, we propose the following research questions (RQ):

RQ1. For student translators, what are the similarities and differences between translation and paraphrasing in terms of the cognitive effort involved at the text level?
1a. Is translation cognitively more demanding than paraphrase? In other words, does translation mode (inter vs. intra) have a significant impact on the participants’ cognitive effort?
1b. Whether and in what way do target language and text genre affect the cognitive effort during translation and paraphrasing? Besides, are these effects, if any, comparable between translation and paraphrasing?
RQ2. For student translators, whether and to what extent does translation differ from paraphrase in terms of attention distribution pattern?

## Methodology

The current study is part of a larger undergoing research project which adopted eye-tracking and key-logging as two major data collection methods for an integrated analysis of reading and writing behaviours during translation and paraphrase. In addition, these real-time data are complemented by qualitative data from post-experiment retrospective interviews and output analysis. Given that the major purpose is to identify similarities and differences of real-time reading behaviours between intra- and inter-lingual translation, we focus on the participants’ eye movement data in the current study.

### Operationalizing cognitive processing via eye movement data

Eye-tracking has been widely incorporated into translation process research to provide insights into the mental process of translators and deepens the understanding of cognitive effort in translational activities [29]. By using well-established eye measures such as average fixation duration and fixation count [30], we attempt to capture the real-time cognitive effort in paraphrase and translation, which informs us about the task feature and the impact of external variables on the participants’ cognitive mechanism.

This study adopts an empirical-experimental design to investigate student translators’ real-time cognitive processing in paraphrase and translation from two perspectives: One is the amount of cognitive effort as measured by average fixation duration and fixation count. Generally, longer fixations and more fixations indicate greater cognitive effort [31]. The other is the attention distribution pattern. It reflects the way in which the cognitive resources are allocated. Attention distribution pattern allows researchers to make inferences on translators’ attention coordination mechanism [32]. Specifically, we examined the participants’ attention shift count between ST and TT areas. This measure refers to the frequency of attention switch between ST and TT, i.e., how many times did participants direct their fixations from ST towards TT or vice versa, which is associated with the cognitive management during translation [31].

### Participants

The study was conducted at the Hong Kong Polytechnic University. Twenty-four postgraduate students majoring in translation and interpreting at colleges in Hong Kong were recruited. The sample consisted of 20 females and 4 males and were aged between 22 and 37 years (*M* = 24.71, *SD* = 3.16). All the students were Chinese native speakers with Mandarin (L1) as their dominant language and English (L2) as their first foreign language. Before the experiment, they had taken compulsory translation courses for at least 12 consecutive weeks and were familiar with translation strategies and skills. All potential participants filled out a questionnaire on their background, including their education experience and self-declared English language proficiency. To ensure their upper-intermediate or advanced proficiency level in English, only those scored 6.5 or higher on the International English Language Testing System (IELTS) exam and/or had obtained a TEM-8 (Test for English Majors-Band 8) certificate were invited to take part in the experiment (TEM8 is a criterion-referenced test for measuring the overall English proficiency of senior undergraduates majoring in English Language and Literature in China). Each participant was paid HKD 200 for their participation.

### Tasks and materials

Three within-subject independent variables were included in the design: translation mode (translation vs. paraphrase) was the primary focus, additionally, to examine the effect of potential factors relating to cognitive processing, we added target language (English vs. Chinese) and text genre (news report and tourism promotional material) as the modulating factors. Thus, each participant carried out four types of tasks (L1-L2 translation, L1 paraphrasing, L2-L1translation, L2 paraphrasing) for the two types of STs.

Eight texts of similar length (ranging from 155 to 170 words) were selected from official online websites. The news reports in both English (EN1 and EN2) and Chinese (CN1 and CN2) were selected from the Bilingual Reading Section on the Financial Times (Chinese) Website (http://www.FTChinese.com). Regarding the tourism promotional texts, the English materials (ET1 and ET2) were chosen from the TripAdvisor Website (https://en.tripadvisor.com.hk/), and the Chinese texts (CT1 and CT2) were picked from the Mafengwo, a Chinese travel SNS website (http://www.mafengwo.cn/). The genre differences are based on the texts’ primary functions and expected readership. According to the text typology by Reiss (1971), news reports can be classified as representational or content-dominated texts, informing facts about specific events (e.g., what, when, who, why). The tourism texts can be categorized as appellative or appeal-dominated, aiming for certain effects on the target reader, for example, turning the reader into an actual tourist. This goal is realized by using specific linguistic strategies such as direct form of address, informal tone and imperative mood [33].

To improve the comparability of the texts, we attempted to match the major textual features of the source materials by revising parts of the text, for example, replacing low frequency words, adjusting the syntactic structures, and shortening long sentences [34]. Table 1 summarizes the textual profiles of the English and Chinese STs separately. To examine whether this textual manipulation would disrupt reading and processing for translation students at post-graduate level, we also recruited four external graders who were PhD students majoring in Translation and Interpreting Studies to evaluate its degree of reading ease, translation difficulty and coherence of each text. The reading ease mainly includes information density, abstractness, and required background knowledge [35]. The raters were asked to read each text and give a score on the mentioned five aspects from 1 (very easy) to 5 (very difficult) on a 5-point Likert Scale questionnaire. They were also welcome to provide any comments on the texts. Minor revisions were made according to their ratings and feedback. We examined whether different texts reveal a significant difference in three indicators of the reading ease and the text coherence using the linear mixed-effects model comparison with the *lme4* package in R [36, 37]. The results confirmed a comparable degree of the reading ease and text coherence among the texts (information density, *p* = .378; abstractness, *p* = .285; required background knowledge, *p* = .160; coherence, *p* = .103). We further conducted pairwise comparisons on the translation difficulty between different experimental texts via the *emmeans* package [38]. No significance was observed in either pair of texts, confirming an equal level of difficulty among all experimental texts (S1 Table).

**Table 1:** Summary of the textual information of English and Chinese STs.

**Chinese ST**				
Text	CN1	CN2	CT1	CT2
Genre	News	News	Tourism	Tourism
Word count	170	164	162	165
Number of sentences	9	10	7	10
Lexical raw type token ratio	9.94	9.40	8.57	9.30
Mean depth of dependency trees	6.89	6.00	6.89	6.5
Raw type token ratio of collocations	6.63	6.33	6.63	6.32
**English ST**				
Text	EN1	EN2	ET1	ET2
Genre	News	News	Tourism	Tourism
Word count	165	158	155	157
Number of sentences	8	9	6	6
Percent of complex words	13.94%	20.25%	12.90%	17.83%
Flesch Kincaid Reading Ease	48.2	48.2	46.3	42
Flesch Kincaid Grade Level	11.7	10.9	13.2	13.9
Automated Readability Index	13.4	12.1	14.6	13.9

Note: The linguistic features in Chinese texts were extracted using L2C-rater [39]. The lexical raw type token ratio reflects the lexical density of the text, and the mean depth of dependency trees is chosen to represent text’s syntactic complexity. The raw type ratio of collocations represents the collocation density in the text. In terms of English texts, Flesch Kincaid Reading Ease uses a score between 1 to 100 to reflect the readability of a text, mainly based on word and sentence length. A higher score indicates lower difficulty in reading a text. This score can be further converted into the Flesch Kincaid Grade Level, which shows the approximate grade level needed for successful comprehension. The Automated Readability Index offers another assessment of the required grade level in America to understand a text, which highlights the role of the character length in words and sentences.

The whole experiment consisted of four blocks, with one task in each block. Each task contained two texts of the same genre (either news or tourism), and the text genre in each task was counterbalanced among the participants. The task order was also counterbalanced across the participants according to the Latin Square Design.

### Equipment

The experiment was programmed by *Tobii Studio 3*.*4*.*8* and the *Translog II*. For the recording of eye tracking data, Tobii TX 300 was used, with a frequency of 300HZ and a viewing distance of 55-65cm from the screen. In addition to the eye movement data, the *Translog II* also registers the participants’ keystrokes, changes and revisions during the translation and paraphrasing. In the present study, we only report results based on eye movements recorded by the *Tobii Studio*.

### Procedure

This experiment has been approved by The Polytechnic Institutional Review Board (IRB) (Ref No. HSEARS20161229004). Participants were tested individually. A written consent form was obtained from each of them before the experiment (S3 Appendix). After that, they first carefully read instructions on the procedures and set-ups. Before each experimental task, the participants went through a set of warm-up practices to familiarize themselves with the eye-tracker, the *Tobii studio* and *Translog* systems, calibration, blind typing, and task requirements. They were allowed to ask any questions after the warm-ups for further explanation. Then they were asked to perform four tasks: two translation tasks from English to Chinese and vice versa, as well as two paraphrasing tasks in English and Chinese. Each task started with a 9-point calibration and validation, followed by a detailed instruction. The instruction explicitly stated the expected function of the output text according to the text genre in this task. For news texts, participants were asked to translate or paraphrase the text to inform the readers of the complete content of the news. For tourism texts, they were expected to produce an appropriate text in a travel brochure to attract the target readers. After that, they went through another 5-point calibration and validation with a faster speed. Then they started the experimental task by pressing a recording button. During each task, the computer screen was divided into two parts, with the ST displayed at the top and the participant’s input at the bottom. No time limits were imposed but participants were encouraged to complete the task within a realistic time frame. In the process of translation and paraphrase, the participants were encouraged not to shake their body and were not allowed to use external resources such as the Internet and electronic dictionary. A list of low-frequency words was provided beforehand to ensure the successful completion of the task. There was a five-minute compulsory break after each task.

Considering the experiment was quite time-consuming and laborious, completing all the tasks within one day was impracticable. Therefore, data collection lasted two days. Each participant was asked to complete two tasks on the first day and finish the remaining two on the second. Retrospective interviews were immediately carried out when participants finished two tasks in a day. During the interviews, the participants watched the replay of the video recordings of their translation and paraphrasing process at 1.5x speed, during which the experimenter paused at any points, asking the participants to explain the reasons or motivations for behaviors that may reflect problems in cognitive processing. The interviews were loosely based on questions eliciting the participants’ perception of the task difficulty, self-judgement of their performance and explanations for their specific operations or strategies. The whole experiment lasted for approximately 4 hours on average.

## Results

### Data filtering and cognitive effort indicators

The gaze sample of all the eye-tracking data reached the baseline of 84%. Adjacent fixations of less than 75s were merged and short fixations of less than 60s were discarded. Each ST and target text (TT) region was set as one AOI (Area of Interest), which was drawn manually according to each participant’s distribution of the fixation points via visual inspection. The rationale for this ST-TT distinction is that fixation data over the source and target texts are different in the type of reading performed by the participant, with different purposes and specific operations. For instance, fixations during TT reading are results not only of target language reformulation but also of “mechanical time-consuming operations related to TT typing [30]”.

To investigate the cognitive effort of four types of tasks, the participants’ average fixation duration (AFD) and average fixation count (AFC) were adopted as indicators. AFD refers to the average duration for each fixation and was exported from the *Tobii Studio* directly. AFC was calculated by dividing the number of total fixations by the number of words in ST. To explore the attention distribution pattern of four types of processing, the attention shifts between ST and TT was adopted as indicators, which was collected based on the visit count data between ST and TT and further rechecked manually.

### Statistical analysis

First, we checked the distribution of the data points in each data set using *qqnorm* and *plot* packages [40] and deleted the outliers accordingly to normalize the dependent variables. The remaining data were further analyzed using linear mixed-effects model. We adopted the maximal random effect structure justified by the data through a forward model comparison approach with a significant value of .20 [41]. Random slopes for interaction predictors which significantly improve the model fit (*p* < .20) were all included in the final model. All the data analyses were performed in R.4.1.1.

### Data results

#### General patterns of translation and paraphrasing

**Fig 1:**
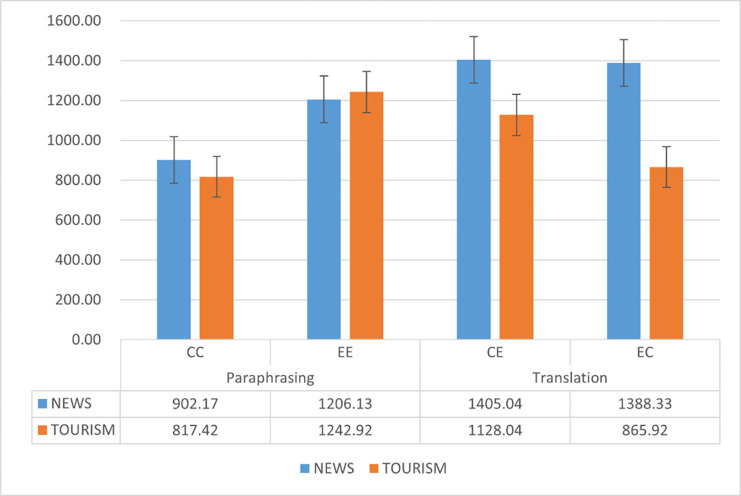
Total task time (s) for translation and paraphrasing in different conditions.

Total time spent for each type of task was averaged among all the participants and summarized in Fig 1. It was found that when the participants worked into their L1, translation always takes longer duration than paraphrasing irrespective of text genre; however, for tasks into L2, the participants spent more time on news translation than on news paraphrasing; but for the tourism texts, they devote longer time for paraphrasing than for translation. This implies a considerable interaction between genre and target language in modulating the cognitive processing of intra- and inter-lingual translation, which need be testified by inferential statistics.

#### Cognitive effort of translation and paraphrasing

**Fig 2:**
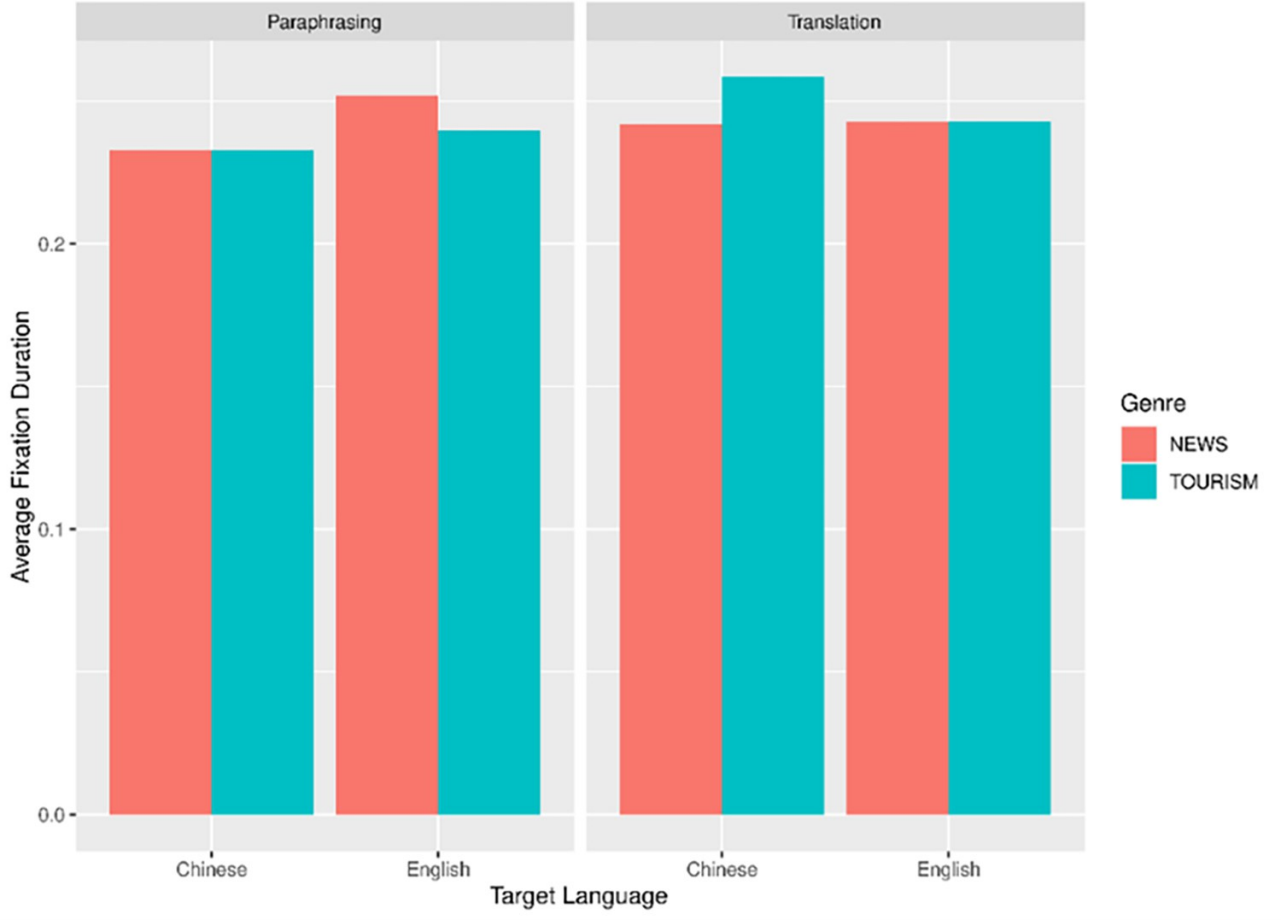
Average fixation duration in ST.

**Fig 3:**
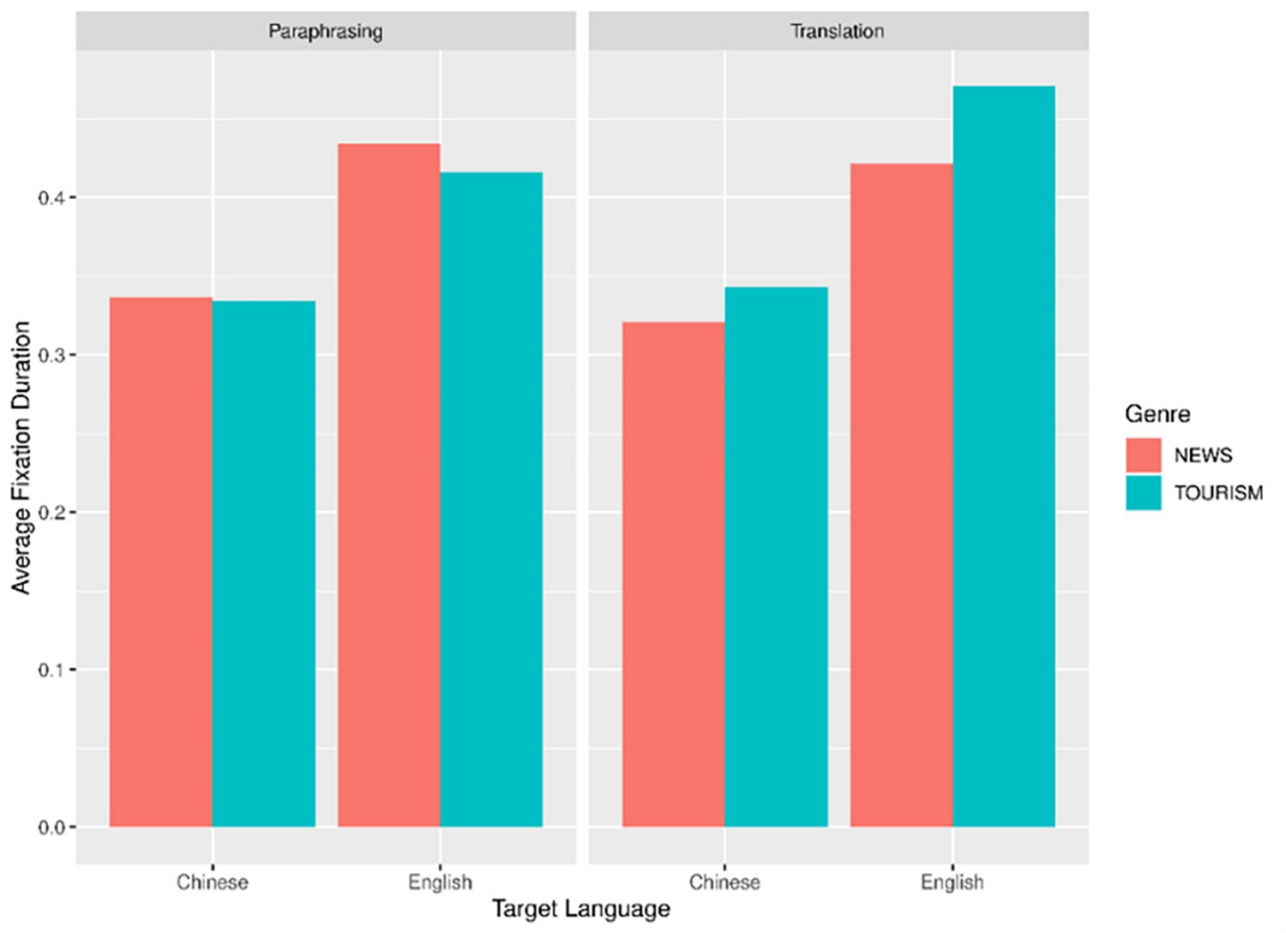
Average fixation duration in TT.

With average fixation duration (AFD) as the dependent variable, we built two linear mixed-effects models for ST region and TT region separately. Both models contained Mode (Paraphrasing vs Translation), Target Language (Chinese vs English), and Genre (News vs Tourism) as interaction predictors, together with random intercepts by Participant and Text. All the interaction predictors were contrast-scaled using the *scale package* [42], and inverse transformation was applied to the AFD data for the Linear mixed-effects model (LMM) analysis. For ease of interpretation, language- and genre-related differences across paraphrasing and translation are visualized by using the package *ggplot2* as shown in Figs 2 and 3 respectively [43].

In ST region, two out of 192 data points were discarded as outliers before statistical analysis (1.04% of all the data). Results of the final model are presented in Table 2. We found a significant main effect of task mode, with longer AFD in the translation task than in the paraphrasing task (*t* = 6.88, *p* < .001). There was also an interaction effect between task mode and target language (*t* = -2.37, *p* = .032). Specifically, L2 paraphrasing resulted in significantly longer AFD in the ST region compared to L1 paraphrasing (*t* = 2.29, *p* = .031). By contrast, L2-L1 translation tended to require longer AFD than L1-L2 translation, but this tendency did not reach significance (*t* = 0.97, *p* = .342).

**Table 2:** Model results for average fixation duration in ST.

	Estimate	Std.Error	df	t-value	Pr (>|t|)
Mode	0.097	0.014	82.77	6.88	< 0.001***
Target language	0.032	0.025	18.07	1.26	0.22
Genre	-0.014	0.028	11.79	-0.5	0.62
Mode: Target language	-0.07	0.03	15.3	-2.37	0.032*
Mode: Genre	-0.04	0.04	13.4	-0.9	0.38
Target language: Genre	-0.06	0.04	12.7	-1.86	0.09
Mode: Target language: Genre	-0.02	0.02	5.39	-0.8	0.46

Model:lmer(TransResults~cMode*cTargetLanguage*cGenre+(cTargetLanguage+cGenre+cMode: cTargetLanguage+cTargetLanguage:cGenre+cMode:cGenre+1|Subject)+(1|Text),data = AFD_ST, REML = T)

In TT region, five outlier data were discarded (2.60% of all the data). Table 3 presents the results of the final model. A significant effect of target language was confirmed (*t* = 11.56, *p* < .001). As shown in Fig 3, the production of English (L2) texts needed considerably longer AFD in TT in both task modes.

**Table 3:** Model results for average fixation duration in TT.

	Estimate	Std.Error	df	t-value	Pr (>|t|)
Mode	0.02	0.02	108.35	1.18	0.24
Target language	0.33	0.03	15.28	11.56	<0.001***
Genre	0.02	0.03	11.57	0.6	0.56
Mode: Target language	0.05	0.02	4.03	1.89	0.13
Mode: Genre	-0.009	0.02	126.72	-0.49	0.62
Target language: Genre	-0.03	0.02	129.59	-1.58	0.12
Mode: Target language: Genre	-0.003	0.03	5.2	0.146	0.89

Model: lmer(TransResults~ cMode*cTargetLanguage*cGenre + (cTargetLanguage+cGenre +1|Subject)+(1|Text), data = AFD_TT, REML = T)

**Fig 4:**
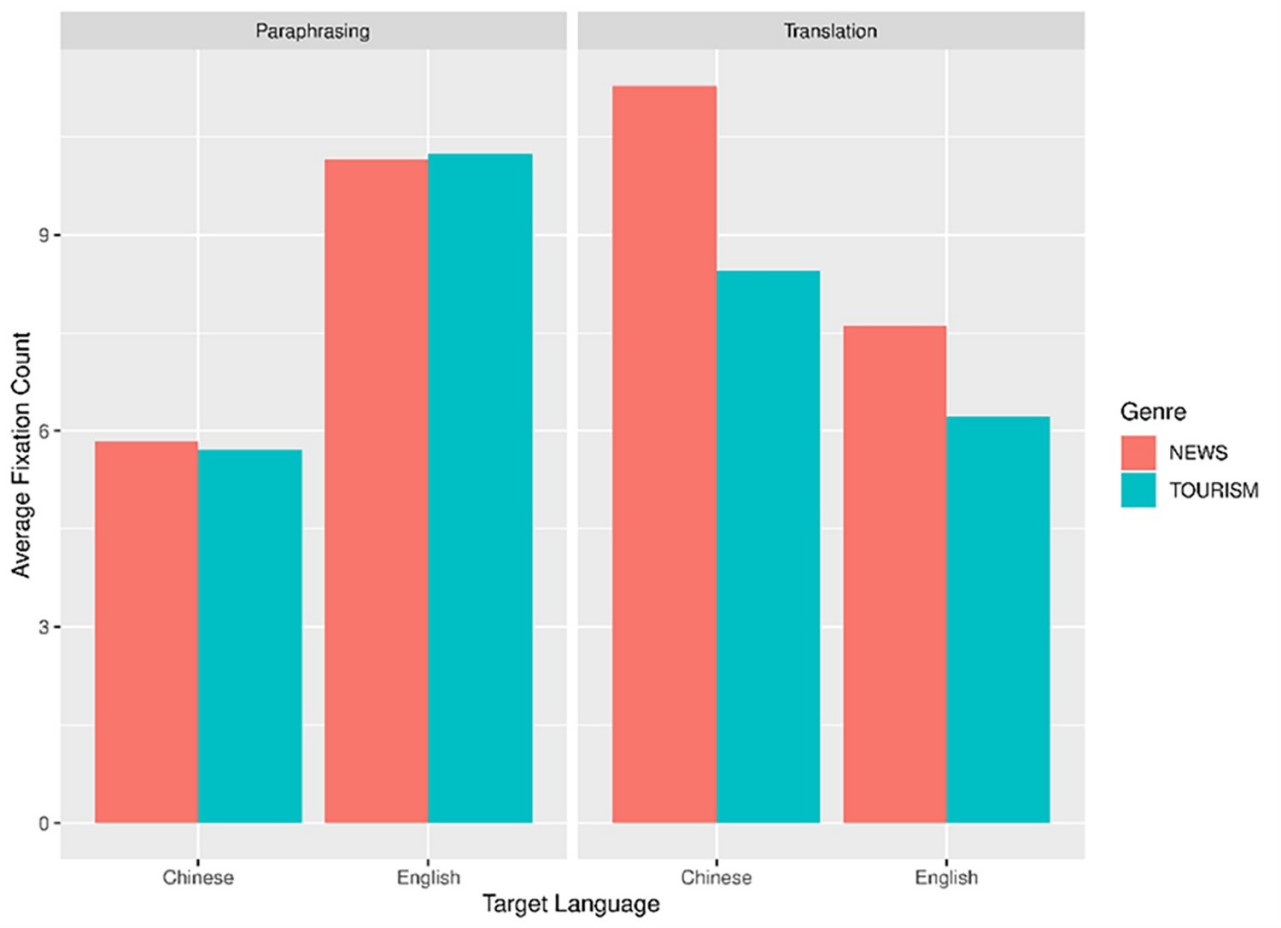
Average fixation count in ST.

**Fig 5:**
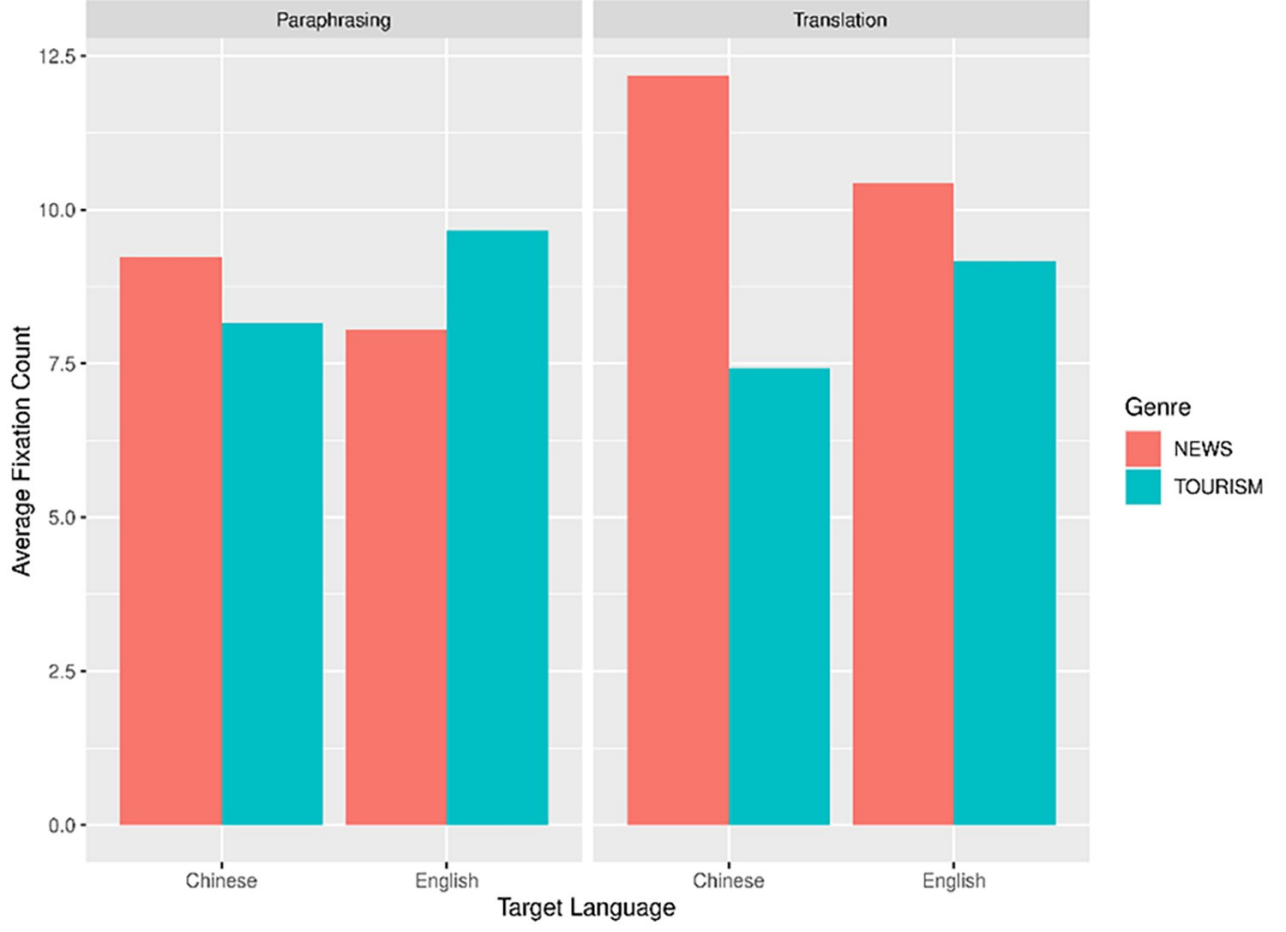
Average fixation count in TT.

Similarly, we built another two linear mixed-effects models for the other dependent variable, average fixation count (AFC). The model construction and contrast scaling were identical to that in the AFD analysis. For the AFC data, we applied log transformation for the LMM analysis. Figs 4 and 5 illustrate the language- and genre-related differences across paraphrasing and translation in ST and TT regions respectively.

Two outlier data were discarded in ST region according to the data distribution (1.04% of all the data). The remaining data were further analyzed using LMM. As presented in Table 4, target language had a significant effect on AFC, with generally more fixations per word in ST in L2 production compared to L1 production (*t* = 2.80, *p* = .011). The interaction of task mode and target language also reached significance (*t* = -10.45, *p* < .001). Further analysis showed that in the paraphrasing task, the AFC in ST in L1 paraphrasing was smaller than that in L2 paraphrasing (*t* = 6.48, *p* < .001); in translation task, there were more AFC in ST in L2-L1 translation compared to L1-L2 translation (*t* = 4.24, *p* < .001). In other words, L2 ST comprehension resulted in significantly more fixations per word in both translation and paraphrasing tasks. Besides, we also found a significant interaction effect between task mode and genre (*t* = -4.29, *p* < .001). Fig 4 illustrates that in the translation task, the AFC in the news ST was distinctly larger than that in the tourism ST. By contrast, in the paraphrasing task, the AFC in ST between two types of text genre was similar. This suggests that the genre effect which played a role in translation task was weakened in paraphrasing.

**Table 4:** Model results for average fixation count in ST.

	Estimate	Std.Error	df	t-value	Pr (>|t|)
Mode	0.01	0.02	21.88	0.75	0.46
Target language	0.07	0.02	18.91	2.80	0.011*
Genre	-0.06	0.03	16.25	-1.74	0.10
Mode: Target language	-0.22	0.02	5.078	-10.45	< 0.001***
Mode: Genre	-0.07	0.02	115.62	-4.29	< 0.001***
Target language: Genre	-0.02	0.03	16.89	-0.55	0.59
Mode: Target language: Genre	0.02	0.02	6.88	0.79	0.46

Model: lmer(TransResults~ cMode*cTargetLanguage*cGenre + (cMode+cTargetLanguage+cGenre+ cTargetLanguage:cGenre + 1|Subject)+(1|Text), data = AFC_ST, REML = T)

In TT region, two data points were deleted as outliers (1.04% of all the data). The results of the LMM analysis were presented in Table 5. A significant effect of mode was confirmed, suggesting that there were considerably less AFC in paraphrasing task than in translation task (*t* = 2.22, *p* = .033). We also found a significant interaction effect between task mode and genre (*t* = -3.36, *p* = .001). Further analysis again confirmed a reduced genre effect in paraphrasing task compared to the translation task: in translation task, the number of fixations per word in the news text was significantly larger than that in the tourism text (*t* = -3.084, *p* = .015), whereas in the paraphrasing task, this difference in text genre did not reach significance (*t* = 0.264, *p* = .794). Additionally, there also existed a significant interaction effect between target language and genre (*t* = 2.51, *p* = .013). The news text required considerably larger AFC than the tourism text when translated/paraphrased into Chinese (L1) (*t* = -2.991, *p* = .017), but this difference disappeared in the production of English (L2) texts (*t* = -0.157, *p* = .878).

**Table 5:** Model results for average fixation count in TT.

	Estimate	Std.Error	df	t-value	Pr (>|t|)
Mode	0.047	0.021	34.11	2.22	0.033*
Target language	0.017	0.034	21.90	0.51	0.62
Genre	-0.077	0.037	10.84	-2.10	0.06
Mode: Target language	0.010	0.027	3.97	0.38	0.72
Mode: Genre	-0.066	0.020	133.09	-3.36	0.001**
Target language: Genre	0.050	0.020	134.36	2.51	0.013*
Mode: Target language: Genre	0.016	0.033	7.69	0.50	0.63

Model: lmer(TransResults~ cMode*cTargetLanguage*cGenre + (cTargetLanguage+cGenre+ cMode:cTargetLanguage:cGenre +1|Subject)+(1|Text), data = AFC_TT, REML = T)

#### Attention distribution of translation and paraphrasing

**Fig 6:**
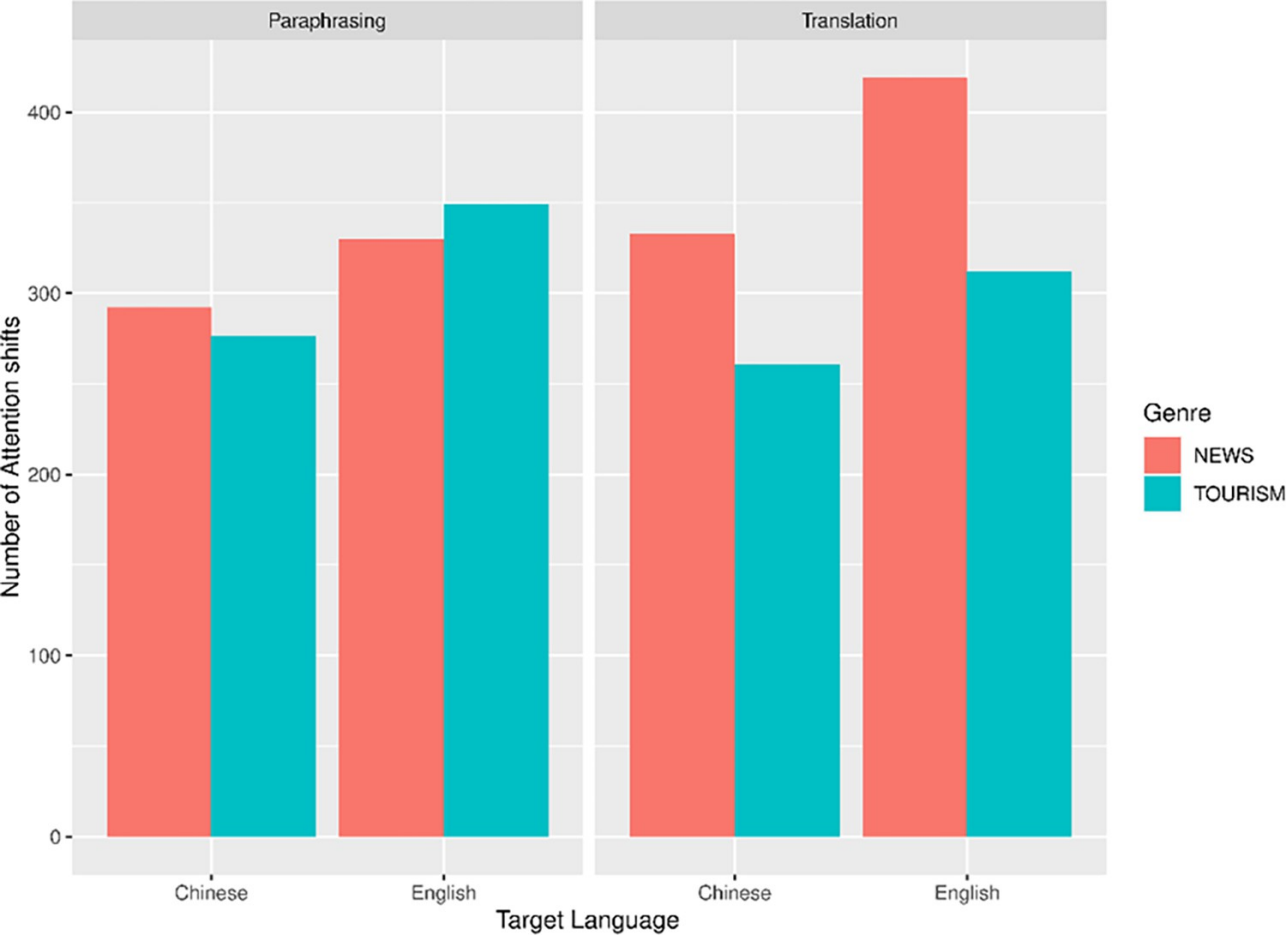
Attention shift between ST and TT regions.

We analyzed the attention shift pattern between ST and TT regions (Fig 6). Seven out of 192 data points were discarded as outliers (3.65% of all the data). Log transformation were applied to the attention shift data and the LMM results were presented in Table 6. We found a pronounced effect of target language, showing that there were more attention shifts in the production of English (L2) than Chinese (L1) in both task modes (*t* = 6.971, *p* < .001). The interaction of task mode and genre also reached significance (*t* = -3.349, *p* = .001). Further analysis revealed that in the translation task, the news text tended to have more attention shifts between ST and TT compared to the tourism text (*t* = -2.033, *p* = .071). However, in the paraphrasing task, the attention shift pattern in two types of text genre was similar (*t* = 0.087, *p* = .932).

**Table 6:** Model results for attention shift between ST and TT regions.

	Estimate	Std.Error	df	t-value	Pr (>|t|)
Mode	0.030	0.024	17.589	1.146	0.27
Target language	0.119	0.017	30.802	6.971	< .001***
Genre	-0.064	0.033	16.894	-1.955	0.07
Mode: Target language	0.003	0.021	5.313	0.152	0.89
Mode: Genre	-0.050	0.015	118.328	-3.349	0.001**
Target language: Genre	-0.0002	0.029	13.480	-0.008	0.99
Mode: Target language: Genre	-0.011	0.022	5.688	-0.488	0.64

Model: lmer(TransResults~ cMode*cTargetLanguage*cGenre + (cMode+cTargetLanguage+cGenre+ cTargetLanguage:cGenre + 1|Subject)+(1|Text), data = AS, REML = T)

## Discussion

This study is one of the first empirically-based attempts to investigate the differences and similarities between intra- and inter-lingual translation in English-Chinese language combination. We are interested in whether translation and paraphrase are cognitively different in the participants’ processing effort. In response to our research questions, we only discuss significant main effects of individual predictors as well as significant pairwise interactions between task mode and the other predictors separately.

### Effect of task mode on cognitive effort

Our discussion started with the data patterns in ST region. The model analysis revealed a considerable effect of task mode on AFD over ST regions. Translation was cognitively more demanding in light of ST reading behaviors, as seen by significantly longer fixation duration irrespective of text genre and target language. As described in section 3.2.2, all the source materials were comparable in their major linguistic properties, which suggests that significant differences during this stage, if any, were not due to intrinsic textual features but the cognitive operations involved in translation and paraphrase. Specifically, although both modes require producing the TT derived from the ST, participants are essentially engaged in different cognitive operations in the stage of reformulation as translation involves bilingual transfer and paraphrasing requires searching for different lexical and syntactic forms [3]. Previous research has confirmed a higher mental cost in translation than in other similar tasks. For example, Shreve et al [44] compared the cognitive effort between reading in anticipation of translation, reading in anticipation of paraphrasing and reading in anticipation of comprehension. It was found that reading for translation elicited longer task time than the other two types of reading and had more problems in the output, implying higher mental costs in translation. A similar comparative approach was taken by Jakobsen and Jensen [45] who investigated the eye movements across reading while typing a written translation, reading in preparation of translation, reading for monolingual comprehension and reading while speaking a translation. The eye-tracking analysis supported the greater cognitive effort involved in reading for translation than in monolingual tasks. Although the tasks in our experiments are not the same to those in the above studies, the findings nevertheless converge in the cognitive burden associated with cross-language transfer which, distinguishes translation from other similar tasks. Therefore, we infer that bilingual transfer in translation elicited significantly greater cognitive effort than synonyms retrieval, sentence restructuring and other operations exclusively involved in paraphrase.

The model analysis also detected a considerable mode effect on AFC in TT region. The participants had significantly more fixations over the target region in translation than in paraphrase. Previous translation process research found that compared to ST reading, TT reading involves more complex cognitive operations as indicated by slightly more fixations [45]. One likely interpretation was that TT reading requires not only reading of the existing and emerging TTs but also the constant visual monitoring of the translation progress [29, 45]. Considering the notably denser fixations in TT region during translation, we infer that the on-line monitoring of translation was cognitively more effortful than that of paraphrase and was characterized by a higher degree of recursiveness [46]. We speculate that the participants in translation were engaged in substantial monitoring and modification of lexical choices and syntactic reformulation than in paraphrase as a result of the mental costs in bilingual transfer. For instance, they may go back numerous times to the previous TT items for a concise and coherent target production.

### Effect of target language and genre on cognitive effort

In consistency with our hypothesis, the model results showed that text genre and target language played a role in shaping the cognitive efforts. As for AFD, there was a significant main effect of target language in TT region as well as significant interaction between mode and target language in ST region. Taken together, we found that L2 language proficiency may play a decisive role in modulating the participants’ cognitive efforts. It is not unreasonable to see L1 production as more automatic, allowing richer vocabulary and more varied syntactic choices. Thus, production in one’s mother language (L1 paraphrase and L2-L1 translation) requires less processing capacity.

Interestingly, this L1 advantage was only partially supported by data on reading behavior in ST region: L2 paraphrase elicited significantly longer AFD in ST region than L1 paraphrase. while the differences between L1-L2 and L2-L1 translation were negligible. A closer examination of the interaction effect reveals that although L2-L1 translation generated longer AFD than L1-L2 translation (see Fig 1), the differences failed to achieve statistical significance. The fact that translation and paraphrase were influenced by target language mode to strikingly different extents might be attributed to the horizontal approach common to inter-lingual translation. Translators are constantly engaged in a horizontal process during which they search and establish links between SL and TL at morphological, syntactic, lexical and semantic levels [47] and this process could occur very early. An eye-tracking study investigation on inter-lingual translation confirmed that translation involved a parallel process in which SL and TL were concurrently activated, in particular, target language structures were anticipated during the ST reading [48]. In this sense, cognitive costs in ST reading during translation mainly arise from both SL encoding and coactivation of TT items and sometimes efforts relating to TT activation may exceed the ones for SL comprehension. Different from translation, paraphrase as monolingual transfer is predominantly a sequential process during which target output takes shape only when source comprehension has been completed. Taken together, it is likely that the facilitation effect due to L1 advantage was override by the extra costs for the cross-linguistic activation and planning during translation.

With regards to AFC, there was a significant language effect on ST region. Analyzing the data separately, we found that AFC in ST regions during L2 paraphrase significantly outnumbered that during L1 paraphrase. Additionally, L2-L1 translation generated significantly more fixations than L1-L2 translation. The pattern combined suggests that ST reading under L2 mode was featured by more frequent and dense fixations between words and lines in search for contextual information, which to some degree reflects the cognitive pressure of L2 decoding irrespective of task mode and text genre.

Text genre also conditioned the cognitive efforts in terms of AFC on both ST and TT regions. There was a significant interaction effect between genre and task mode in both ST and TT region. The model reported similar AFCs between news text and tourism text during paraphrase. This genre effect was strengthened during translation with more AFCs for news text than for tourism texts. One tentative explanation for this may be that the participants adopted genre-specific strategies more frequently in translation than in paraphrase. They may have higher requirements for the faithfulness and accuracy of news which contained factual information, thus devoting more fixations for monitoring and modification. As introduced in section 1.2, paraphrase was seldom taught as a core sub-skill of translation, neither had it been incorporated into students’ daily training. Consequently, the participants were less sensitive to genre-specific differences when paraphrasing and may merely focus on rewording of linguistic forms.

### Effect of target language and genre on attention shift

Our last question addresses the participants’ attention distribution between ST and TT regions. Model results demonstrate that translation and paraphrase did not differ significantly in numbers of attention transitions. It seemed that the participants adhered to similar processing flows in translation and paraphrase. In addition, we found that shifts of visual attention occurred more frequently in L2 production than in L1 production as reported by a significant main effect of target language. This echoed our speculation in section 5.2 that participant’s cognitive processing was constrained by their L2 proficiency to a greater extent than by task mode or text genre. Lastly, a significant interaction between task mode and genre indicated that the effect of task mode on attention shift counts depended heavily on genre of the ST. Similar to previous findings on AFC, no considerable differences in shift occurrence were found between news text and tourism text in paraphrase while this gap was widened between the two genres in translation. A significant genre effect under inter-lingual task mode suggested participants were more acquainted with genre-specific strategies and quality standards for translation due to regular training and practice, leading to different attention allocation modes between paraphrase and translation.

## Conclusion

This study explored the cognitive process in translation and paraphrase with a focus on student translators’ reading behaviors. The eye movement data offers empirical evidence for the cognitively more demanding feature of inter-lingual transfer than its intra-lingual counterpart. It was also found that the mode-induced effect on the cognitive effort and attention distribution were conditioned by text genres and target language, which reveals that giving attention to these modulating factors can be used for a more comprehensive understanding of intra- and inter-lingual translation. Moreover, a notable effect of target language reveals the impact of L2 proficiency in constraining student translators’ cognitive processing during both translation and paraphrase, which highlights the importance of bilingual knowledge as a subcomponent of translation competence. Lastly, although translation and paraphrase differed in the amount of cognitive effort during on-line processing, some features were overlapped such as the attention distribution pattern, implying that procedures and skills can be transferable between the inter and intra mode. In other words, despite the different cognitive operations involved in translation and paraphrase, both modes require an efficient process of message conceptualization and reformulation, a crucial component of translation. Thus, regular practice in paraphrase would to some degree facilitate development of translation competence.

The study has several limitations. First, the data analysis distinguished ST section between TT section but did not further classify different types of reading involved in these sections, for example, TT reading may involve three types of sub-activities including TT reading, TT reformulation and TT typing. Second, translation process in real-life practice could be constrained by a variety of linguistic and contextual variables, which have not been fully considered in the current design. Finally, the study examined only student translators with no between-group comparison with professional translators, who may exhibit difference reading and processing patterns.

## Supporting information

S1 Appendix Experimental materials. (PDF)

S2 Appendix Low frequency words list. (PDF)

S3 Appendix Sample of participation consent. (PDF)

S1 Table Pairwise comparison results on the translation difficulty of experimental texts. (PDF)

S1 Dataset Experiment data. (XLSX)

S1 File Data analysis for eye-tracking experiment. (R)
