# Supplementary material for: A cognitive inquiry into similarities and differences between translation and paraphrase: Evidence from eye movement data
Source: PLoS One. 2022 Aug 5;17(8):e0272531. doi: 10.1371/journal.pone.0272531 (PMC9355232; doi:10.1371/journal.pone.0272531)
Supplement: S1 Appendix — (PDF) [file pone.0272531.s001.pdf]

Experiment materials

CN1
周轶君曾是一名国际记者。成为母亲后，她渐渐发现祖辈代代相传、习以为常的育儿经验正越来越不足以应对社会的飞速发展。所置身的教育体制暴露出种种问题，但转向缓慢，困难重重。在家和孩子“斗争拉锯”、思考为孩子选择什么样的教育路径，复杂性堪比当年的巴以局势。过去的价值不再适用，既有的体系遭到质疑，我们该去哪里找“参考答案”？于是她决定拍一部教育主题的纪录片《他乡的童年》，向更广阔的世界寻求解答。日本、芬兰、英国、印度，最后回到中国，她把这部片子形容为“一个母亲在路上”。放下成见，带着问题，回归细节，用影像捕捉对一国教育的感知，大量日常的细节在镜头里流泻出来。这个片子能让焦虑之中的中国的家长们看到，在地球的另一个地方，教育还可以是这样的。(170)

CN2
刚刚过去的 2020 年已成为历史上最热的年份之一。最新研究显示，随着全球气候变暖，去年全球平均气温比 1850 年至 1900 年期间的平均气温高出约 1.2 摄氏度。2020 年里，一系列与气候变化有关的异常事件和自然灾害接踵而至，包括北大西洋创纪录的飓风季节，以及西伯利亚地区北极的大热浪。联合国秘书长 Guterres 对此发表措辞严厉的讲话。他警告大家，世界正在走一条“自杀性的”道路。“大自然已经在以日益强大的力度和愤怒进行反击，”他表示，“灾难性的火灾和洪水、热带气旋和飓风日益成为新常态。“ 他呼吁停止使用煤炭，并促请所有国家制定净零排放目标。未来 10 年至关重要。到 2030 年，全球人为造成的二氧化碳净排放量必须下降约 45%。与自然和平相处是 21 世纪的标志性任务，它必须成为世界各国的当务之急。(164)

CT1
隐藏、穿插在钢筋水泥、高楼大厦之间的胡同，是北京人内心深处的记忆。在北京能够称之为胡同的有上千条，纵横交错，遍布城区。他们已有 800 多年的历史，是百姓休养生息的场所，也是北京历史文化发展演变的重要舞台。所谓胡同，是由一排排比邻的四合院串联组成的小街道。乍一看它们都是矮矮的、灰灰的，然而这里却蕴含着众多传奇般的经历和趣闻掌故。要体会老北京的味道，胡同游是必不可少的行程，自行车则是最好的交通工具。您可以找出扔在车棚的自行车，带着轻松愉悦的心情在胡同里自由穿行。近几年，越来越多的酒吧和时尚小店悄悄出现在这些小街上。在北京城最古老的斜街里，各种特色的小店、餐馆、酒吧热闹非凡。(165)

CT2
巴厘岛是印度尼西亚最为著名的旅游休闲区，被许多旅游杂志评选为世界上最令人陶醉的度假目的地之一。巴厘岛南部拥有迷人的白色沙滩及温暖的阳光。众所周知，阳光与海滩是巴厘岛的“招牌”。而受地形及洋流的影响，巴厘岛多数海滩海浪较大，虽不适合游泳，却是绝佳的冲浪地点。对于喜欢户外活动的人来说，巴厘东部及北部并不比南部逊色，相反更加吸引他们。西部是潜水圣地，有 30 米长靠近海滨的珊瑚墙。北部则是一个适合徒步的地方，占主导地位的是两座有名的活火山。不少人从四面八方赶来观看它们超凡脱俗的美丽。传统的巴厘舞蹈与岛上的绘画、音乐、雕刻一样遐迩闻名。在这里，舞蹈不是为了娱乐，而是常常成为祭祀的一部分，是一种与神沟通的形式。(165)

EN1
A train crash in eastern Taiwan killed dozens of people, in what could be the province’s deadliest rail disaster. The train travelling from Taipei derailed close to Qingshui, a scenic stretch of coastline where marble cliffs drop into the Pacific Ocean. According to the transportation ministry, a construction truck operated by the railway administration slid into

the track from a worksite on the hillside and collided with the train. Leaders of nearby countries extended their sympathies to the victims and offered assistance to the authorities. The area where the crash occurred is well-known for hazardous traffic conditions. Eastern Taiwan is blocked off by towering mountain ranges and the only road linking the main eastern city of Hualian to the north passes sheer cliffs with narrow curves in several places. As the lightly populated east is popular with tourists, many people travel by train to avoid mountain roads. Fortunately, an improved road bypassing some of the most dangerous sections with tunnels was opened one year ago. (165)

EN2
It has been ten years since a tsunami laid waste the Pacific coast of Japan. The tsunami and the undersea earthquake which triggered it threw the lives of tens of millions into anxiety. The Fukushima reactors melted after this tsunami knocked out their cooling systems. Water subsequently used to cool the reactors became polluted with radioactive nuclides. In Shanghai and San Francisco iodised salt jumped off the shelves as people looked for prevention of which they had no need. Recently, Japan announced that they will construct equipment to release polluted water into the Pacific, which has been condemned by environmentalists, fishermen and neighbouring countries. This decision risked reviving some of the trauma of the nuclear accident and worsening its legacy of pollution. However, Japanese authorities argued that there was no practical alternative to releasing the water as storage space ran out. They added there was no risk to human health and discharges will start in about two years. (158)

ET1
France is the country where iconic tourist attractions like the Eiffel Tower meet charming French countryside, and splendid grandeur of châteaux can only compete with the lavishness of the Cote D'Azur resorts. The country of wine, seductive language and romantic citizens who elevate their culinary fantasies to the level of obsession is perhaps one of the most popular tourist destinations in the world drawing like a magnet all sorts of travelers from singles, to couples to anyone in between. With so much to do and see, our France travel tips below come in handy. Lyon is one of the gastronomic cities in France and is believed to be the place where cinematography emerged. The city is best explored by foot, by renting a bike or using public transportation. Wander along the narrow streets of Old Lyon to remind yourself how it all started taking in all the incredible architecture, lovely restaurants and various grocery stores (155)

ET2
Tourists to South Korea can expect a country proud of its cultural roots as well as a country blessed with beautiful topographical areas that only add to the diversity to be had within its many cities and towns. The capital of Seoul is home to less than 10 million people. Travelers in this capital city can visit the National Museum of Korea where over 220,000 items are on display, including a historical gallery and an outdoor exhibit. Fine arts, calligraphy, clothing and food exhibits are also found here, to give the vacationer a wide range of Korea's history and cultural significance through a variety of mediums. Travelers to South Korea are met with many wonderful things to behold, and some that may be more thought provoking than others, such as the Adult Sculpture Park. Definitely not for the shy or easily embarrassed, this park allows adults to walk amid the sculptures for a different type of attraction. (157)
