# Supplementary material for: A cognitive inquiry into similarities and differences between translation and paraphrase: Evidence from eye movement data
Source: PLoS One. 2022 Aug 5;17(8):e0272531. doi: 10.1371/journal.pone.0272531 (PMC9355232; doi:10.1371/journal.pone.0272531)
Supplement: S2 Appendix — (PDF) [file pone.0272531.s002.pdf]

Low frequency words list

CN1	巴勒斯坦 Palestine	以色列 Israel
CN2	热带飓风 tropical cyclone	煤炭 coal
	飓风 hurricane	摄氏度 Celsius
	大西洋 Atlantic	西伯利亚 Siberia
CT1	水泥 cement	
CT2	洋流 ocean current	珊瑚 coral
	祭祀 worship	地形 landform
EN1	Taipei 地名，台北	Qingshui 地名，清水（台湾省）
	derail v. （列车）脱轨	marble adj. 大理石的
	hazardous adj. 有危险的	
EN2	tsunami n. 海啸	Fukushima 地名，福岛（日本）
	radioactive nuclide 放射性核素	iodised adj. 碘化的
ET1	Eiffel Tower 埃菲尔铁塔	grandeur n. 庄严、壮丽、宏伟
	Châteaux n. 城堡，古堡	lavishness n. 浪费、过度
	Cote D’Azur resorts 蔚蓝海岸度假胜地	culinary adj. 烹饪的
	gastronomic adj. 烹饪的，美食的	seductive adj. 诱人的，诱惑的
	Lyon 城市名，里昂	cinematography n. 电影摄影术
ET2	topographical adj. 地形的	behold v. 注视，看
