# Supplementary material for: A cognitive inquiry into similarities and differences between translation and paraphrase: Evidence from eye movement data
Source: PLoS One. 2022 Aug 5;17(8):e0272531. doi: 10.1371/journal.pone.0272531 (PMC9355232; doi:10.1371/journal.pone.0272531)
Supplement: S3 Appendix — (PDF) [file pone.0272531.s003.pdf]

THE HONG KONG POLYTECHNIC UNIVERSITY
香港理工大學

參與研究同意書

A Comparative Enquiry into Cognitive Processes of Translation and Paraphrasing --- Evidence from Eye-tracking and Key-logging
翻譯和改寫任務中認知過程的比較研究 --- 來自眼動與按鍵記錄的證據

本人____________________同意參與由___李德超老师团队____開展的上述研究。

本人知悉此研究所得的資料可能被用作日後的研究及發表，但本人的私隱權利將得以保留，即本人的個人資料不會被公開。

研究人員已向本人清楚解釋列在所附文件上的研究程序，本人明瞭當中涉及的利益及風險；本人自願參與研究項目。

本人知悉本人有權就程序的任何部分提出疑問，並有權隨時退出而不受任何懲處。

參與者姓名 ____

參與者簽署 ____

研究人員姓名 ______________________________________

研究人員簽署 ______________________________________

日期 ____

Hung Hom Kowloon Hong Kong 香港 九龍 紅磡
Tel 電話 (852) 2766 5111 Fax 傳真 (852) 2784 3374
Email 電郵 polyu@polyu.edu.hk
Website 網址 www.polyu.edu.hk
