# Supplementary material for: A cognitive inquiry into similarities and differences between translation and paraphrase: Evidence from eye movement data
Source: PLoS One. 2022 Aug 5;17(8):e0272531. doi: 10.1371/journal.pone.0272531 (PMC9355232; doi:10.1371/journal.pone.0272531)
Supplement: S1 Table — (PDF) [file pone.0272531.s004.pdf]

Table. Pairwise comparison results on the translation difficulty of experimental texts.

contrast	estimate	SE	df	z.ratio	p.value
CN1-CN2	-1.1747	0.856	Inf	-1.373	0.8697
CN1-CT1	-0.0696	0.890	Inf	-0.078	1.0000
CN1-CT2	-1.5926	0.855	Inf	-1.864	0.5761
CN1-EN1	-0.2627	0.910	Inf	-0.289	1.0000
CN1-EN2	-0.6810	0.882	Inf	-0.772	0.9945
CN1-ET1	-0.4263	0.974	Inf	-0.438	0.9999
CN1-ET2	-1.1018	0.977	Inf	-1.128	0.9509
CN2-CT1	1.1051	0.873	Inf	1.265	0.9116
CN2-CT2	-0.4179	0.828	Inf	-0.505	0.9996
CN2-EN1	0.9120	0.896	Inf	1.018	0.9718
CN2-EN2	0.4937	0.863	Inf	0.572	0.9992
CN2-ET1	0.7484	0.959	Inf	0.780	0.9941
CN2-ET2	0.0728	0.958	Inf	0.076	1.0000
CT1-CT2	-1.5230	0.872	Inf	-1.746	0.6568
CT1-EN1	-0.1931	0.930	Inf	-0.208	1.0000
CT1-EN2	-0.6114	0.903	Inf	-0.677	0.9976
CT1-ET1	-0.3567	0.994	Inf	-0.359	1.0000
CT1-ET2	-1.0323	0.996	Inf	-1.037	0.9689
CT2-EN1	1.3299	0.893	Inf	1.489	0.8135
CT2-EN2	0.9116	0.859	Inf	1.061	0.9646
CT2-ET1	1.1663	0.957	Inf	1.219	0.9266
CT2-ET2	0.4908	0.954	Inf	0.514	0.9996
EN1-EN2	-0.4183	0.843	Inf	-0.496	0.9997
EN1-ET1	-0.1636	1.001	Inf	-0.163	1.0000
EN1-ET2	-0.8391	1.004	Inf	-0.836	0.9911
EN2-ET1	0.2547	0.975	Inf	0.261	1.0000
EN2-ET2	-0.4209	0.975	Inf	-0.431	0.9999
ET1-ET2	-0.6756	1.039	Inf	-0.650	0.9981

Note: The data of the ratings can be found in ‘Translation difficulty rating’ in ‘S1_Dataset.xlsx’. The translation difficulty of each experimental text contains ratings of every sentence in this text (e.g. The first sentence in CN1 is marked as ‘CN1S1’) as well as the overall translation difficulty of the whole text (e.g. The overall translation difficulty of CN1 is marked as ‘CN1O’).
